# Open source and reproducible and inexpensive infrastructure for data challenges and education

**DOI:** 10.1038/s41597-023-02854-0

**Published:** 2024-01-02

**Authors:** Peter E. DeWitt, Margaret A. Rebull, Tellen D. Bennett

**Affiliations:** 1 Department of Biomedical Informatics, University of Colorado School of Medicine, University of Colorado, Aurora, CO USA; 2 Section of Critical Care Medicine, Department of Pediatrics, University of Colorado School of Medicine, University of Colorado, Aurora, CO USA; 3 Children’s Hospital Colorado, Aurora, CO USA

**Keywords:** Paediatric research, Paediatrics, Research data

## Abstract

Data sharing is necessary to maximize the actionable knowledge generated from research data. Data challenges can encourage secondary analyses of datasets. Data challenges in biomedicine often rely on advanced cloud-based computing infrastructure and expensive industry partnerships. Examples include challenges that use Google Cloud virtual machines and the Sage Bionetworks Dream Challenges platform. Such robust infrastructures can be financially prohibitive for investigators without substantial resources. Given the potential to develop scientific and clinical knowledge and the NIH emphasis on data sharing and reuse, there is a need for inexpensive and computationally lightweight methods for data sharing and hosting data challenges. To fill that gap, we developed a workflow that allows for reproducible model training, testing, and evaluation. We leveraged public GitHub repositories, open-source computational languages, and Docker technology. In addition, we conducted a data challenge using the infrastructure we developed. In this manuscript, we report on the infrastructure, workflow, and data challenge results. The infrastructure and workflow are likely to be useful for data challenges and education.

## Introduction

Data sharing is necessary to maximize the actionable knowledge generated from research data. The FAIR (Findability, Accessibility, Interoperability, and Reusability) principles^1^ are NIH-supported^2^ guidelines for scientific data management and stewardship. Data Challenges, can encourage secondary analyses of datasets and facilitate development of high-quality decision support tools using those data. They are also common in computational training programs.

Data challenges in biomedicine can require considerable computational resources, which may be provided by cloud computing infrastructure. However, cloud computing infrastructures can be expensive and may require industry partnerships to conduct the challenge. Examples include challenges run by PhysioNet^3^ that use Google Cloud virtual machines and “Dream Challenges” hosted by Sage Bionetworks (https://sagebionetworks.org/research-projects/dream-challenges-powered-by-sage-bionetworks/). Such robust infrastructures can be financially prohibitive for investigators without substantial resources. Given the potential to develop scientific and clinical knowledge and the NIH emphasis on data sharing and reuse, there is a need for inexpensive and computationally lightweight methods for data sharing and hosting data challenges. We conducted this study to fill that gap.

We developed a workflow to share prospectively collected clinical study data and to host a data challenge with the goal of optimizing use of the study data. We then conducted the Harmonized Pediatric Traumatic Brain Injury (HPTBI) Data Challenge. The workflow allows for reproducible model training, testing, and evaluation. To accomplish this, we leveraged public GitHub repositories, open-source computational languages, and Docker technology.

## Methods

### Clinical problem

Pediatric traumatic brain injury (TBI) is the cause of approximately 35,000 hospitalizations and 2,200 deaths per year in the United States^4^. Non-mortality sequelae of severe TBI affect both the child and the child’s family due to cognitive impairment, decreased physical and mental health, and lower quality of life^5–7^. Interventions to improve outcomes for pediatric severe TBI patients may have substantial beneficial impact on the child, family, and community.

Although TBI is known to be an important public health problem, few recommended treatments have a strong evidence base^8^. One reason for the limited evidence base is the lack of access to, and utility of, clinical TBI datasets.

### Data Source: Prospective PEDALFAST study

The data supporting the HPTBI data challenge were collected in the NICHD-funded PEDiatic vAlidation oF vAriableS in TBI (PEDALFAST) multi-center prospective cohort study. PEDALFAST was conducted at two American College of Surgeons (ACS)-certified level 1 Pediatric Trauma centers between May 2013 and June 2017. PEDALFAST was the first study to report the use of the functional status score (FSS) outcome measure^9^ in a particular patient population^10^. In addition, PEDALFAST supported the development of computable phenotypes for accurately identifying neurosurgical and critical care events in administrative data^11^. Important PEDALFAST variables include demographics, injury mechanism and severity scores, interventions and treatments, neurologic exams, encounter information, and patient outcomes. The objective of the HPTBI data challenge was for participants to build reproducible models to predict (1) hospital mortality and (2) the FSS at hospital discharge for critically injured children with TBI. The study was approved by the IRBs of the institutions where data was collected. In addition, the Colorado Multiple Institutional Review Board approved the conduction of the data challenge and the dissemination of these data.

PEDALFAST data enrolled 395 subjects, of which 388 had adequate data quality to be de-identified and made public. Inclusion criteria for the study were (1) age less than 18 years at the time of hospital arrival, (2) a diagnosis of acute TBI, defined using well-established criteria as “an alteration in brain function or other evidence of brain pathology caused by an external force with possible or suspected trauma,”^12^ (3) admission to an intensive care unit (ICU), and (4) Glasgow Coma Scale (GCS) score of 12 or less documented by a trauma surgery, ICU, or emergency department (ED) attending physician or a neurosugical procedure in the first 24 hours hospital admission. Exclusion criteria were discharge from the ICU within 24 hours of ICU admission without a surgical or critical care intervention such as ventilation (invasive or non-invasive), intracranial pressure (ICP) monitoring, operative procedure, arterial or central venous catheter, or osmolar therapy, or death.

PEDALFAST captured demographic (age, sex, etc.), injury (mechanism, severity scroe, abbreviated injury scale, etc.), and clinical event information (neurosugical procedures, ICU procedures, osmolar therapy, inotropes/vasopressors, ED and ICU GCS and components scores, etc.)^10,11^ on eligible patients. A complete listing of the variables along with simple summary statistics are provided the “datasets” vignette of the pedalfast.data R package^13^ and as part of the example repository^14^.

#### FITBIR

First, we retrospectively mapped the study data to the NIH-supported common data elements (CDEs) used by the Federal Interagency TBI Research (FITBIR) Informatics System (https://fitbir.nih.gov) and submitted the data to that system. The mappings we developed between the PEDALFAST data dictionary and the FITBIR schema are reproducible and publicly available at (https://fitbir.nih.gov/study_profile/395). We posted the dataset on the FITBIR system, where approved users can access and download the data.

We mapped the PEDALFAST data to FITBIR CDEs and made it publicly available in September 2020. We mapped most of the PEDALFAST data to elements of standard FITBIR forms for Demographics, Injury History, Imaging Read, Functional Status Scale (FSS), Surgical and Therapeutic Procedures, Neurological Assessment: Glasgow Coma Scale (GCS), and Pupils. We created one additional, study specific form, to report ICP monitor placement and durations (TBI Vital Signs_EPO from^15^). We also contributed two unique data elements to FITBIR. PEDALFAST recorded pupil reactivity as ‘both reactive’, ‘one fixed’, ‘both fixed’, or ‘unknown.’ Prior to our submission, the data elements in FITBIR for pupil reactivity were left/right eye specific. The data element PupilReact was created to capture the PEDALFAST recorded pupil reactivity. Additionally, prior to our submission there was no good way to report if the eyes were obstructed, making GCS assessment difficult. The data element EyesObscuredInd as created to recorded this information within FITBIR.

#### R Data Package

In order to further lower barriers to data reuse, we also shared the de-identified data in comma separated value (csv) format in a public R data package (https://CRAN.R-project.org/package=pedalfast.data) and Zenodo^14^. This data format is more comparable to the original data collection forms and is likely to be more familiar to potential data users without extensive informatics training. The R data package is publicly available on the Comprehensive R Archive Network (CRAN), the most widely used archive of R packages. In order to avoid inadvertent public availability of test set data during the data challenge, we delayed release of the R data package until the data challenge was complete.

The source code for package development is maintained behind institutional firewalls in order to protect the PHI in the original study dataset. Some of the methods needed for mapping the data to the FITBIR standard are provided in the R data package as functions. For example, we provided a function for rounding patient ages to the FITBIR standard.

In addition, we provided methods for quickly encoding numerically collected ordinal or categorical data as factors. For example, the Glasgow Coma Scale (GCS) motor response is provided as integer values 1 through 6. These six levels correspond to specific categorical values: (1) no response/flaccid, (2) abnormal extension to pain, (3) abnormal flexion to pain, (4) withdraws from painful stimuli, (5) localizes pain or withdraws to touch, and (6) obeys commands. The function pedalfast_factor provides a quick and standardized method for mapping the integer values to a labeled factor for any relevant variable in the provided data set. Specific methods for GCS and FSS categories are provided for ease of use.

### Data challenge

#### Data challenge participant workflow

The workflow we developed was inspired by the PhysioNet/Computing in Cardiology Challenges^3^. We advertised the data challenge on Twitter, Facebook, Slack, and through direct emails to mathematics, computer science, and statistics departments at regional universities. We allowed several weeks for participants to register using a Google Form. We provided labeled data for 300/388 subjects as the training data set, as well as a data dictionary. A holdout test set of the remaining 88 subjects was used for evaluation. The train/test split was balanced between the data collection sites. We provided a template repository on GitHub (https://github.com/cuamc-dop-ids/hptbi-hackathon) and Zenodo^14^ that included a skeleton for working with either R or Python. Participants needed to fork this repository and provide the hackathon administrator with read/write access to the fork. They would then personalize the description.yaml file with contact information and a brief summary of their models, develop their models using R or Python, and expand upon the included simple Docker file.

The provided Docker file defined a simple image. Directions and examples were provided to to participants so they could extend the Docker files to build images with the needed system and language prerequisites required by their model training and fitting methods. We also provided template files for preparing data sets and defining each model and a prediction method for each model as well as infrastructure files so that participants could test their code within a Docker container. The only difference between participant testing and data challenge evaluation was access to the testing data partition.

Participants used another Google Form to alert the data challenge administrator that a submission was ready to be evaluated. Participants were allowed multiple submissions to verify that the code would run to completion on the test set. Only error/successful run status was returned to the participant on developmental submissions. Participants were allowed one final submission.

#### Data challenge assessment

Each submission was identified by a tag in the forked repository. We evaluated submissions on a 2018 MacBook Pro with 16GB RAM and an Intel Core i9 processor.

In an effort to minimize the amount of work that the administrator needed to do for each submission we wrote a bash script, taking two arguments (participant’s github user id and submission version number) to automate all assessment steps while providing verbose error messaging. Specifically, the bash script fetched and merged the participant submission into the submodule used to track the participant’s developement and submissions, created needed branches, insured only certain files have been edited (sha256 checks), ran the training and testing code in a Docker image, generated appropriate results, and pushed the assessment to the participant’s repository. A skeleton version of the administor’s repository, which used git sub-modules to manage participant submissions, is available via Zenodo^14^.

The evaluation scripts first checked that the length of the prediction vectors were complete: 300 for the training set and 88 for the testing set. FSS predictions were required to be complete for the known FSS values in the dataset: 251 and 68 for the training and testing sets respectively. In addition, the FSS predictions were required to be integer valued inclusively between 6 and 30 and the mortality predictions were required to be in a character-valued vector with elements “Mortality” and “Alive.”

We evaluated mortality predictions using the Matthew’s correlation coefficient (MCC) and the F1 score. We evaluated FSS (integers between 6 and 30) predictions using the mean squared error (MSE) between observed and predicted values. Because the data challenge evaluation process including training the models using the participants’ code, we evaluated each model 100 times to verify consistency (reproducibility) in the results. For each participant, we calculated the mean and standard deviation of each assessment statistic and its ranking. We ranked submissions based on (1) accuracy (MSE for FSS, MCC and F1 for mortality), (2) reproducibility (SD of MSE, F1, and MCC over repeated assessments), and (3) model parsimony. For each participant, outcome, and dataset, we calculated the average rank over the mean value and the standard deviation of the assessment statistics. The sum of the average ranks for each participant, outcome, and dataset became the “outcome rank.” This ranking approach tended to select the submitted models with reproducible results, as those models had assessment statistic standard deviations of zero and a shared rank of 1. The overall data challenge ranking was based on the sum of all assessment statistic rankings from both models. Ranking ties were broken by assigning the minimum value: if there was a three way tie for second place, the vector of ranks would be^1,2,5,6^. Cash prizes were provided to the top 3 participants.

#### Anonymization of participants

For this manuscript we have anonymised the participants by assigning an identifier of the form P01, P02,… with the digits derived by alphabetizing the hash of the github user id.

## Results

### Submissions and language choice

Overall, 27 participants completed the registration form. Of these, 11 (40.7%) provided a final submission. Most (8/11) participants used R and 3/11 used Python. Failed submissions most often occurred when participants neglected to provide specific tags and a corresponding version number in a description file. These were necessary to facilitate the use of automated evaluation scripts. The data challenge administrator was often able to quickly provide feedback on these issues, and subsequent submissions were successful. Other reasons for failed submissions included when Docker files were not updated by participants to include the Python modules or R packages required by their code. These errors were also readily identifiable by the data challenge administrator and feedback was provided to allow participants to make corrections and resubmit.

### Models

Each of the eleven participants submitted two models for a total of 22 submitted models. Random Forest was the most common modeling approach (10/22, with many participants using different methods for mortality and FSS prediction), but participants also used unpenalized linear (3/22) and logistic (3/22) regression, ridge (1/22) regression, support vector machines (1/22), gradient boosting (2/22), and stacked models (2/22), Table 1.

**Table 1:** Programming languages and modeling approaches used by each participant for the Harmonized Pediatric Trauma Brain Injury Data Challenge.

Participant	Language	FSS	Mortality
P01	R	Random Forest	Random Forest
P03	R	Gradient Boost	Gradient Boost
P07	R	Linear model	Logistic Regression
P11	python	Random Forest	Support Vector Machine
P12	R	Linear model	Logistic Regression
P14	R	Random Forest	Random Forest
P15	python	Ridge Regression	Random Forest
P20	R	Linear model	Logistic Regression
P22	R	Stacked Models	Stacked Models
P24	python	Random Forest	Random Forest
P26	R	Random Forest	Random Forest

The three linear models used for FSS modeling used a Gaussian Response.

### Data quality and predictor variables

Most submissions used an automated data-driven approach to feature selection. For example, one participant iteratively fit models and selected the predictor sets for both FSS and mortality models using backward-stepwise elimination, cross-validation, and variable selection based on variance inflation factors. Another participant selected variables using the R package VSURF (variable section using random forests)^16^.

Only one of the 11 submissions included code which clearly indicated that work was done to mitigate inconsistent or illogical data values. For example, there are records with the time from admission to start/end of an ICP monitor are inconsistent with hospital length of stay, (Table 2).

**Table 2:** Example of inconsistent data values.

Data Set	Hospital	Admission to	Admission to
Data Set	LOS	ICP Monitor Start	ICP Monitor Stop
Training	20	60	68
Training	6	0	10
Training	20	0	36
Training	1	0	2
Testing	8	91	2
Testing	1	0	2

The Hospital length of stay (LOS) in days is less than the number of days from admission to placement or end of ICP monitoring. This type of inconsistent data observation was present in both the training and testing partitions of the data set.

We also identified common issues in prediction model development including the use of inappropriate predictor variables. For example, participants were instructed that FSS was assessed at hospital discharge, and therefore was only available for survivors and should not be used to predict mortality. The presence of an FSS assessment was an indication of a hospital disposition other than mortality. Nevertheless, one participant was disqualified for including FSS in a mortality prediction model and 3/11 participants used hospital disposition (which includes mortality and is collected simultaneously with FSS) to predict FSS.

### Missing data

Participants dealt with missing data in several different ways. One participant used the R package mice (multivariate imputation by chained equations)^17^ to impute missing values within the data sets. Another participant built models to use missing values as informative. One submission used Python’s OneHotEncoder ignoring missing (unmapped) values and/or dropped missing values. Code reviews for three submissions yielded no clear accounting for missing data.

The most common approach, used by at least five submissions, was to replace missing values with a dummy variable. In most cases this was done by assigning the numeric value zero to missing values. This approach may have led to well-performing models for the data challenge but render the models useless in a clinical application or in understanding of the associations between the predictors and outcomes.

For example, the GCS is qualitative in nature, but is commonly reported as an integer value and treated as a quantitative variable in models. Higher numeric values indicate better neurological status. The minimum score in any of the three GCS categories (eye, motor, verbal) is 1 and thus the minimum total GCS is 3. Replacing missing GCS values with a zero and analyzing GCS as a quantitative variable in the models implied a more severe injury than the most severely injured patients with a known GCS score had.

#### Model performance - testing versus training data

**Fig. 1:**
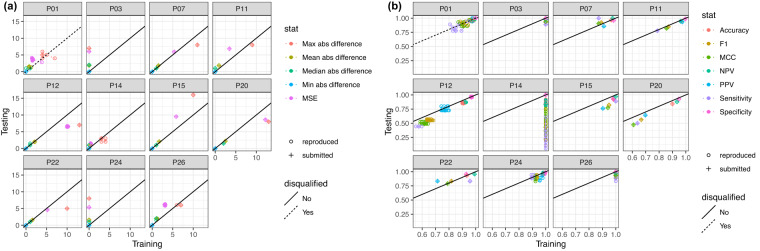
Model Performance on Testing versus Training Data for (**a**) FSS model and (**b**) mortality model.

Model performance on the testing and training datasets is shown in Fig. 1 for the FSS and Mortality models, respectively.

For the FSS model, participants P24 and P03 had zero error on the training set. However, their performance was worse on the held-out testing data, likely indicating over-fitting. For the mortality model, P26, P14, and P03 also likely over-fit their models.

#### Ranking

**Fig. 2:**
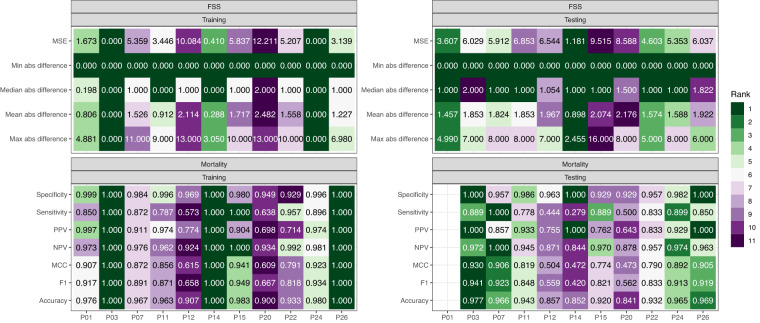
Mean value of Assessment Statistics and Participant Ranking.

The ranking of submissions was based on (1) accuracy (MSE for FSS and MCC and F1 for mortality), (2) reproducibility (standard deviation of MSE, FSS, and MCC over repeated assessments), and (3) model parsimony. The overall data challenge rankings were based on a combination of these 3 parameters and on the categorical assessment of clinical utility. Inclusion of clinical utility did not change the overall final ranking shown in Figure 2.

**Fig. 3:**
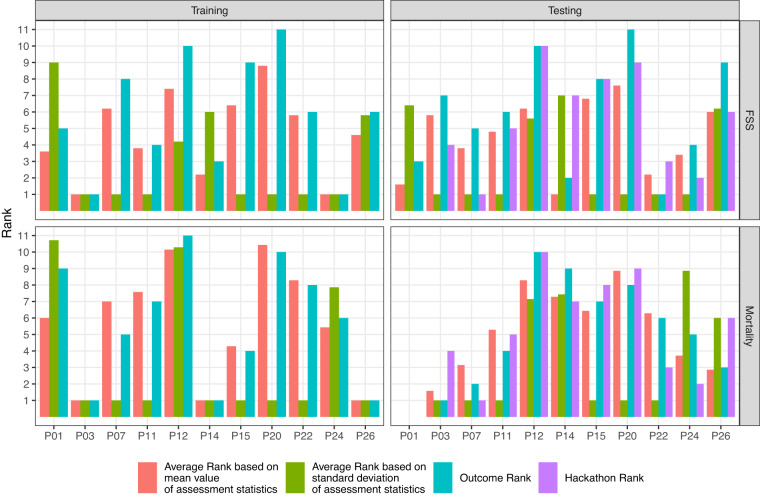
Data Challenge Ranking.

Figure 3 shows the relative ranking for each model and dataset. The overall data challenge ranking is for each model with the ranking in the test set used to award prize money. Table 3 reports the data challenge rank, prize ranking, and prize money.

**Table 3:** Data Challenge Ranking and Prize Money for the top 10 participants.

participant	Data Challenge Rank	Prize Rank	Prize Money
P07	1	1	$500
P24	2	2	$250
P22	3		
P03	4	3	$125
P11	4	3	$125
P26	4	3	$125
P14	7		
P15	8		
P20	9		
P12	10		

P22 was ineligible for prize money for administrative reasons.

### Winning models

The overall winner, P07, used relatively simple models: a Gaussian response linear model for FSS and logistic regression for mortality. This is in comparison to the more complex machine learning methods used by many other participants. The mortality model was found via backward stepwise elimination and, after inspecting the variance inflation factors, focus was given to cardiac arrest (at any time between injury and discharge). The final model used only five predictors, (1) cardiac arrest, (2) age, (3) GCS in the ICU, (4) was mannitol ordered (yes/no)?, and (5) did the patient receive enteral nutrition (yes/no).

The FSS model was also identified via backward stepwise selection, which yielded a 24 variable model. Further variables were removed based on cross validation within the training data, resulting in a final model with only 14 predictors: (1) GCS Eye (ED), (2) GCS sedated (ED), (3) CT skull fracture, (4) CT intra ventricular hemorrhage, (5) GCS Motor (ICU), (6) GCS eye observation (ICU), (7) days from hospital admission to second ICU admission, (8) days from hospital admission to extubation, (9) days from hospital admission to removal of first ICP monitor, (10) days form hospital admission to placement of third ICP monitor, (11) receipt of a new gastrostomy, (12) receipt of a decompressive craniectomy, (13) days from hospital admission to lumbar drain, and (14) hospital length of stay.

## Discussion

We have developed and shared a workflow for data challenges and data challenges that uses open-source, inexpensive, reproducible, and computationally lightweight tools. These methods have the potential to increase the impact of shared research data. In addition, this approach may be useful in computational training programs, as data challenge-type exercises are popular and effective components in many courses.

### Data sharing

Data challenges can encourage secondary use of research data. In the data challenge, the PEDALFAST data were provided in a easy to import and use.csv format. We opted for this format for the data challenge to lower the overhead required for participants to acquire and use the data. Additionally, this allowed us to split the data into development and verification sets. The full data set is available from FITBIR^18^, and as the R package pedalfast.data^13^, and on Zenodo^14^ (10.5281/zenodo.8400499).

With respect to FAIR principles^1^, the Findability and Accessibility of the PEDALFAST data have been achieved via documentation and uploads to persistent public resources, Zenodo^14^ and the Federal Interagency TBI Research (FITBIR) Informatics System. The FITBIR submission meets the Interoperability FAIR principles as the data standard the PEDALFAST data was mapped into will allow for the PEDALFAST data to be easily incorporated into any other TBI research project suppored by data sets housed within FITBIR. Lastly, the Reusability principle of FAIR is shown in use within FITBIR and as the supported data set for a data challenge.

An additional example of reuse: one research group that did not participate in the data challenge used only the publicly available training data to conduct a mortality analysis very similar to one of the data challenge tasks^19^.

### Data challenge

The process was, in general, successful. Multiple participants, models, and submissions were handled by a single administrator using a typical laptop computer. However, there are some important lessons learned which impact the utility of the submitted models.

For advertising and recruitment of data challenge participants, we sent emails to computer science, data science, and statistic departments at regional universities, and also posted on social media. Future data challenge administrators may be able to recruit more participants then we did by using well established workflows and platform, e.g., Kaggle (https://www.kaggle.com) where a community of prospective participants already exists.

With hindsight, it seems that the focus on model performance may have encouraged participants to ignore, or minimize, parsimony, interpretability, and feasibility of implementation. This resulted in most of the submitted models to have limited utility outside of the given data set. Notably, the simplistic mapping of missing values to 0 resulted in values outside the observable range, e.g., GCS. Participants also did not consistently explore the data and consider the relative timing of events (e.g., length of stay and time to ICP monitor placement/removal). This limited the potential clinical impact of some model submissions.

Importantly, no guarantees were made with respect to the analysis readiness of the datasets. Although these data were collected prospectively and are quite clean compared to, for example, electronic health record data, inconsistencies were present in the data. Computational investigators are trained to explore, examine, and prepare data prior to analysis, but we observed that several participants did not do this.

Allowing for and encouraging participants in the data challenge to explore the data and address inconsistencies and missing data is not easily supported by some infrastructures. For example, all competitions hosted on Kaggle require “a clean and well-labeled [sic] dataset.” By design, we did not provide a clean and well-labelled data set and thus required the development of our specific workflow and infrastructure.

The workflow for assessing submissions and returning an evaluation to participants was built and tested to require the data challenge administrator to run a single bash script with a few command-line arguments. However, this script had many failure points with respect to required actions for participants. Some of the failure points could likely be eliminated in future versions of the workflow. For example, requiring version numbering in description files and git-tags could likely be deprecated.

Other potential failure points should be considered when organizing a data challenge. For example, we opted to use docker to define the needed software, system dependencies, etc., for model training and testing. Participants were provided with minimal docker files to define the needed images. In practice, some participants were unable to modify their docker files appropriately and required administrative support. This level of support might not have been possible if the number of participants in the data challenge was, for example, an order of magnitude higher. A single, larger, image with all common modules and packages for all submissions might have been easier for both administrators and participants. The primary image could be extended as needed for edge case modeling.

Overall, contributing the PEDALFAST data to the FITBIR database and as an R data package on CRAN provide multiple paths for interested researchers to find the data set and use it in their work. This is consistent with the FAIR principles.

Data challenges can be run using open-source, reproducible, inexpensive, and computationally lightweight methods. These methods have the potential to increase the impact of shared research data.

## Data Availability

The PEDALFAST^13,14,18^ data is available from the Federal Interagency TBI Research (FITBIR) Informatics System at https://fitbir.nih.gov/study_profile/395. Additionally, the PEDALFAST data is available as an R data package available from the Comprehensive R Archive Network (CRAN) at https://CRAN.R-project.org/package=pedalfast.data and archived on Zenodo at 10.5281/zenodo.8400499.

## Code availability

We provided participants with a template repository on GitHub (https://github.com/cuamc-dop-ids/hptbi-hackathon) that included a skeleton for working with either R or Python. The R package, the participant template repository, and administrator repository have been uploaded to Zenodo^14^ (10.5281/zenodo.8400499) as well.
